# TRIM32 promotes neuronal ferroptosis by enhancing K63-linked ubiquitination and subsequent p62-selective autophagic degradation of GPX4

**DOI:** 10.7150/ijbs.106690

**Published:** 2025-01-20

**Authors:** Xin Zhou, Yuqing Zhao, Shixue Huang, Haoming Shu, Yinuo Zhang, Haiyuan Yang, Yilong Ren, Xuhui Zhou, Wei Liu, Tengfei Song, Jianquan Zhao, Jun Ma

**Affiliations:** 1 Department of Orthopedics, Changzheng Hospital, The Second Affiliated Hospital of Naval Medical University, Shanghai 200003, China.; 2 Department of Orthopedics, Shanghai General Hospital, Shanghai Jiao Tong University School of Medicine, Shanghai 200080, China.; 3 Translational Research Centre of Orthopedics, Shanghai General Hospital, Shanghai Jiao Tong University School of Medicine, Shanghai 200080, China.; 4 Department of Neurosurgery, Changzheng Hospital, The Second Affiliated Hospital of Naval Medical University, Shanghai 200003, China.

**Keywords:** neuronal ferroptosis, TRIM32, GPX4, ubiquitination, autophagic degradation

## Abstract

Ferroptosis, characterized by iron-dependent phospholipid peroxidation, is recognized as one of the cell death pathways activated following spinal cord injury (SCI). However, the precise regulatory mechanisms governing this process remain poorly understood. Here, this study identified TRIM32, an E3 ubiquitin ligase, as a key enhancer of neuronal ferroptosis. TRIM32 promoted neuronal ferroptosis by accelerating the degradation of GPX4, which is an essential inhibitor of ferroptosis. Conditional deletion of *Trim32* in neurons markedly inhibited neuronal ferroptosis and promoted neuronal survival, eventually improving mouse locomotor functional recovery after SCI. However, overexpression of *Trim32* showed aggravated neuronal loss and poor behavioral function, which could be attenuated by ferroptosis inhibitor Liproxstatin-1. Mechanistically, TRIM32 interacted with GPX4, promoted K63-linked ubiquitination modification of GPX4 at K107, thus enhanced p62-dependent autophagic degradation of GPX4. Moreover, ROS-ATM-Chk2 signaling pathway phosphorylates TRIM32 at S55, further contributing to GPX4 ubiquitination and degradation and subsequent neuronal ferroptosis after SCI, suggesting a positive feedback loop between ROS and TRIM32. Clinically, lipid peroxidation was significantly promoted in patients with SCI. These findings reveal that TRIM32 functions as a neuronal ferroptosis enhancer which is detrimental to neuronal survival and locomotor functional recovery in mice after SCI by promoting K63-linked ubiquitination and subsequent p62-dependent autophagic degradation of GPX4, suggesting a promising therapeutic target for SCI.

## Introduction

Spinal cord injury (SCI) stands as a profoundly debilitating condition, often resulting in persistent motor and sensory impairment with a disappoint prognosis^1^, ^2^. SCI arises from an irreversible primary injury, succeeded by a cascade of events known as secondary injury^3^. During this phase, heightened oxidative stress, driven by an overproduction of reactive oxygen species (ROS), profoundly decreases neuronal viability, thus impeding subsequent regeneration and functional recovery^4^, ^5^. Despite notable progress in both fundamental and clinical SCI research, the translation of these findings into effective treatments for promoting functional recovery remains limited due to a failure to maintain functional neuronal survival and axonal regeneration. Hence, unraveling the intricate molecular and cellular mechanisms underpinning SCI and preventing neuronal loss becomes imperative.

In recent years, a growing body of evidence has recognized the significance of ferroptosis, an emerging type of non-apoptotic regulated programmed cell necrosis^6^. This process operates through the accumulation of intracellular iron-induced ROS and subsequent lipid peroxidation^7^. Ferroptosis has been implicated in pivotal roles across diverse realms, including tumor biology, inflammation, immunity, and neuro-disorders^6^, ^7^. Particularly, neurons exhibit inherent vulnerability to oxidative stress due to the abundance of polyunsaturated fatty acid residues within their phospholipid membrane and their limited capacity for scavenging ROS^8^-^10^. Consequently, the strategic targeting of neuronal ferroptosis emerges as a promising strategy for promoting neuronal survival and preserving neurological function following SCI^11^, ^12^.

Tripartite motif containing 32 (TRIM32), a member of TRIM family and an E3 ubiquitin ligase, contains an N-terminal RING domain, one B-box domain, one coiled-coil region and six C-terminal NHL repeats^13^. TRIM32 has been reported to be play a role in various biological processes including innate immune, cell proliferation and glycolysis^14^-^16^. It has been reported that TRIM32 could catalyze ubiquitination to target substrates including p53, c-Myc, STING, ARID1A and TRIF^15^-^19^. However, its role in neuronal ferroptosis, especially in the context of SCI, and its downstream substrates is still unknown and remains to be explored.

Here, we identified TRIM32 as an enhancer of neuronal ferroptosis that promotes GPX4 ubiquitination and degradation. Mechanistically, TRIM32 promoted K63-linked ubiquitination of GPX4 at K107, thereby enhancing p62-GPX4 interaction and leading to p62-dependent selective autophagic degradation of GPX4. Furthermore, ROS-ATM-Chk2 signaling pathway phosphorylates TRIM32 at S55, further contributing to GPX4 ubiquitination and degradation and subsequent neuronal ferroptosis after SCI, suggesting a positive feedback loop between ROS and TRIM32. These findings indicated that TRIM32-mediated ubiquitination promoted GPX4 degradation via p62-dependent selective autophagy to promote ferroptosis after SCI, providing a promising therapeutic strategy for SCI treatment.

## Materials and Methods

### Animals

WT C57BL/6J mice were purchased from the animal center of Naval Medical University. *Trim32^fl/fl^* mice, with a C57BL/6J background (Strain No. S-CKO-14655), were acquired from Cyagen (Suzhou, China). H11-Syn1-iCre mice (Strain No. T052699) were obtained from GemPharmatech (Nanjing, China). To induce conditional *Trim32* deletion specifically in spinal cord neurons, *Trim32^fl/fl^* mice were crossed with *Syn1*^Cre^ mice. All mice of both sexes at eight weeks old were utilized in the experiments. The animals were housed in a facility maintaining a 12-hour light-dark cycle, with appropriate levels of humidity and temperature. They were provided with food and water ad libitum throughout the duration of the study. All experimental procedures were conducted in accordance with the guidelines and approved by the animal committee of Changzheng Hospital, the Second Affiliated Hospital of Naval Medical University.

### Human samples

For the diagnosis of SCI resulting from acute trauma, both neurological assessment and MRI were employed. Patients devoid of neurological or neoplastic disorders, who were scheduled for intrathecal anesthesia, were enlisted as controls. Prior to any procedures, informed consent was obtained from all participants. The study protocols received approval from the ethics committees at Changzheng Hospital, the Second Affiliated Hospital of Naval Medical University. CSF was collected within 48 hours post-injury via lumbar puncture, following established procedures^20^. Simultaneously, blood samples were obtained intravenously. The levels of MDA were then assessed in both serum and CSF using the MDA assay kit (ab118970, Abcam), following the manufacturer's instructions.

### SCI model

To induce a severe spinal cord contusion injury at the thoracic level (T10), a standardized procedure was followed, consistent with previous descriptions^21^, ^22^. Initially, eight-week-old mice were anesthetized via inhalation of isoflurane. Subsequently, a laminectomy was performed from T9 to T11 to expose the spinal cord. Using the Infinite Horizon Spinal Cord Impactor system (IH-0400, Precision Systems and Instrumentation), a controlled impact force of 85 kdyn was applied directly to the spinal cord at the T10 level. Following injury, the muscles and skin were meticulously sutured in layers. To prevent infection, mice received intramuscular injections of penicillin. Post-operatively, bladder was emptied twice daily for mice undergoing T10 contusion injury until normal urination function was restored. For evaluating neuronal ferroptosis *in vivo*, Liproxstatin-1, at a dosage of 10 mg/kg/day, was administered intraperitoneally to the mice for a duration of seven consecutive days following SCI.

### Behavioral evaluation

BMS scoring system was used to assess hindlimb locomotor performance by two trained observers who were unaware of the experimental group conditions. The BMS score, ranging from 0 to 9, was determined based on several locomotor parameters including ankle movement, paw position, trunk stability, and stepping coordination in mice placed in an open field. Any discrepancy of more than two score points between the left and right hindlimbs resulted in the exclusion of the animal from the analysis.

In the hindlimb kinematic analysis, mice underwent overground walking on a runway measuring 60 cm in length and 4 cm in width. During locomotion, bilateral leg kinematics were recorded using an iPhone operating at 60 frames per second. Landmarks, affixed to the crest, hip, knee, ankle joints, and distal toe, were tracked to capture the movement dynamics accurately. In instances where hindlimb movement was limited, the gait was determined based on forelimb motion. Subsequently, recorded videos were subjected to analysis using DeepLabCut to automatically identify and trace the landmarks. This automated process facilitated the precise quantification of various kinematic parameters, including maximal crest and toe height, stride length, and joint angle oscillation, for each gait cycle. To visualize and further analyze the kinematic data, specific parameters were selected to generate a chronophotograph and construct a stick view representation of hindlimb movements using MATLAB.

In the rotarod test, an accelerating rotarod apparatus capable of increasing rotational speed from 0 to 40 r.p.m was employed to assess hindlimb function in mice following SCI. Each mouse underwent one practice trial, succeeded by two test trials, with a 20-minute interval between each trial. The latency to fall was determined by calculating the average from the two test trial for each mouse.

### EMG test

MEPs were evaluated 8 wpi using EMG tests (Shanghai Yuyan Instruments Co., Ltd., China). Briefly, mice were anesthetized with isoflurane inhalation, and spinal cord re-exposure was performed via laminectomy. The stimulation electrode was positioned at the rostral ends of the exposed spinal cord, while the recording electrode was inserted into the mid-belly of the medial gastrocnemius muscle. A grounding electrode was placed subcutaneously. MEPs were induced with a single stimulus (0.5 mA, 0.5 ms, 1 Hz), and the resulting waveforms were recorded and their amplitudes quantified.

### Immunofluorescence staining

For histological analysis, mice were euthanized and underwent perfusion with saline followed by 4% paraformaldehyde. The spinal cords at the injury site were dissected and underwent gradient dehydration in 20% and 30% sucrose solutions for two days before being embedded in OCT compound (Tissue-Tek, Japan). Slices of spinal cord tissue, 20 μm in thickness, were then sectioned and stored at -20°C. Before staining, the sections were washed with phosphate-buffered saline and then incubated with a blocking buffer containing 5% BSA and 0.3% Triton-100 for 1 hour at room temperature. Subsequently, the sections were incubated overnight at 4°C with primary antibodies diluted in a buffer containing 1% BSA and 0.3% Triton-100. Following this, secondary antibody incubation was performed for 2 hours at room temperature. Finally, the samples were imaged using a THUNDER Imaging System (Leica, Germany).

### Cell culture and reagents

For the primary neuron culture, neonatal mouse cortices were promptly excised and sliced into 1-mm^3^ fragments. These tissue pieces were then subjected to enzymatic digestion using 0.125% trypsin and 0.5 mg/mL DNase for 20 minutes at 37°C in a shaking incubator. Following digestion, the cell suspensions were passed through a 70-μm nylon mesh. Subsequently, the neurons were seeded onto dishes pre-coated with poly-L-lysine and cultured utilizing the B-27™ Plus Neuronal Culture System (A3653401, Thermo Fisher) under standard conditions of 37°C with 5% CO_2_. To maintain cell viability and support growth, half of the culture medium was refreshed every two days.

HEK293T cells were obtained from Cell Bank of the Chinese Academy of Sciences and cultured in DMEM supplemented with 10% fetal bovine serum, 100 U/mL penicillin, and 100 μg/mL streptomycin at 37°C with 5% CO_2_.

The reagents used in the study included the following: MG132 (S1748, Beyotime), 3-MA (HY-19312, MedChemExpress), CQ (HY-17589A, MedChemExpress), Baf A1 (HY-100558, MedChemExpress), NH4CI (HY-Y1269, MedChemExpress), CHX (HY-12320, MedChemExpress), Rapamycin (HY-10219, MedChemExpress), Erastin (SC0224, Beyotime), Liproxstatin-1 (HY-12726, MedChemExpress), Ferrostatin-1 (HY-100579, MedChemExpress), H_2_O_2_ (30%, v/v, 7722-84-1, Sigma-Aldrich), and NAC (S0077, Beyotime).

### Neuronal OGD/R injury

An OGD/R injury was performed as previously described in primary neurons^23^. Briefly, the primary neurons were cultured in glucose-free medium at 37°C with mixed gas (94%N_2_/5% CO_2_/1% O_2_) for 60 minutes. Afterwards, the medium was changed with normal neuronal medium and primary neurons were cultured under standard conditions of 37°C with 5% CO_2_. Then analysis was performed after culturing for 2 hours.

### Cell viability and cell death

Primary neuron cell viability was evaluated using CCK-8 assays (CK04, Dojindo) and analyzed using an absorbance microplate reader (ELx800, Bio-Tek). Cell death in primary neurons was assessed using calcein-AM/ethidium homodimer-1 staining (Live/Dead assay, L3224, Thermo Fisher) following the manufacturer's protocols and imaged using a THUNDER Imaging System (Leica, Germany).

### ROS and lipid peroxidation evaluation

ROS levels in primary neurons were assessed using an ROS assay kit (S0033S, Beyotime) via flow cytometry following the manufacturer's instructions. Additionally, lipid peroxidation products MDA and 4-HNE levels in primary neuronal cell lysates were evaluated utilizing lipid peroxidation detection kits (MDA: ab118970, Abcam; 4-HNE: ab238538, Abcam) according to the manufacturer's guidelines.

### Plasmids and adenovirus transfection

Primary neurons were transfected with adenovirus with shRNA-control (shNC) and shRNA-TRIM32, shRNA-ATG5, shRNA-Beclin 1, shRNA-p62, shRNA-ATM or shRNA-Chk2; and adenovirus TRIM32 or adenovirus GPX4 (GenePharma, China). The sequences were as follows: shTRIM32, 5'-GCCGCAAGGAAATTCTCCATT-3'; shATG5, 5'-ATGGCCTCTGACCTTCTACTT-3'; shBeclin 1, 5'-CCCTATGGAAATCATTCCTAT-3'; shp62, 5'-GAGGTTGACATTGATGTGGAA-3'; shATM, 5'-CCGTGGAGATTTCTCAATCTT-3' and shChk2, 5'-CCTTCGTAAATACCGAGCTTA-3'. Full-length sequences for human TRIM32, GPX4, p62, LC3, ubiquitin, and truncation mutants mentioned above were generated using PCR amplification and cloned into Flag-/HA-/His-/Myc-/GFP-tagged vectors. Lipofectamine 3000 reagent (L3000001, Invitrogen) was used for transfection according to the manufacturer's guidelines.

### Adeno-associated virus infection

The pAAV-PHP.eB-hSyn-TRIM32-3×FLAG-WPRE (AAV-Trim32) was constructed to facilitate exogenous TRIM32 *in vivo* (Obio Technology, China), while pAAV-PHP.eB-hSyn-MCS-WPRE served as the control vector (AAV-Con). Two weeks before SCI, the AAV-PHP.eB vector diluted in 0.9% sodium chloride to a final volume of 250 μL with a final titer of 3 × 10^11^ viral genome was injected through the tail vein as previously described^23^.

### RNA isolation and reverse transcription-quantitative PCR (qPCR)

Total RNA extraction from primary neurons was conducted using RNAiso Plus reagent (Takara, Japan), according to the manufacturer's instructions. Following RNA purification and quantification, the total RNA samples underwent reverse transcription into cDNA utilizing the HiScript II Q RT SuperMix for qPCR kit (Vazyme, China). Subsequently, qPCR was carried out using the TB Green® Premix Ex TaqTM kit (Takara, Japan) in an ABI 7900HT qPCR system (Applied Biosystems, Thermo Fisher). Relative expression of *Gpx4* mRNA was normalized to *Actb* and quantified using the 2^-ΔΔCT^ method. The primer sequences for *Gpx4*, and *Actb* were as follows: *Gpx4* primer: forward 5ʹ-GCCAAAGTCCTAGGAAACGC-3ʹ and reverse 5ʹ-CCGGGTTGAAAGGTTCAGGA-3ʹ; and *Actb* primer: forward 5ʹ-GAGCTGCGTTTTACACCCT-3ʹ and reverse 5'-GCCTTCACCGTTCCAGTTTT-3ʹ.

### Western blotting

For total protein extraction, the cells were homogenized in RIPA lysis buffer (KeyGEN BioTECH, China). Following degeneration at 95°C for 10 min, equivalent protein samples were loaded into SDS-PAGE gels for separation and then transferred to the PVDF membranes. The membranes were then blocked with 5% skim milk and incubated with primary antibodies at 4°C overnight. After washing with TBST three times, the membranes were incubated with HRP-conjugated secondary antibodies at room temperature for 1 h and the protein bands were visualized using ECL reagent (Tanon, China). The primary and secondary antibodies included the following: rabbit anti-TRIM32 (10326-1-AP, Proteintech); rabbit anti-GPX4 (30388-1-AP, Proteintech); mouse anti-GPX4 (67763-1-Ig, Proteintech); rabbit anti-ACSL4 (22401-1-AP, Proteintech); rabbit anti-xCT (26864-1-AP, Proteintech); rabbit anti-NRF2 (16396-1-AP, Proteintech); mouse anti-β-actin (66009-1-Ig, Proteintech); rabbit anti-ubiquitin (10201-2-AP, Proteintech); rabbit anti-ATG5 (10181-2-Ig, Proteintech); rabbit anti-Beclin 1 (11306-1-AP, Proteintech); rabbit anti-LC3 (14600-1-AP, Proteintech); rabbit anti-p62 (18420-1-AP, Proteintech); mouse anti-Flag (66008-4-Ig, Proteintech); mouse anti-Myc (60003-2-Ig, Proteintech); mouse anti-HA (66006-2, Proteintech); mouse anti-His (66005-1-Ig, Proteintech); mouse anti-GFP (66002-1-Ig, Proteintech); anti-mouse IgG (A7028, Beyotime); anti-rabbit IgG (A7016, Beyotime); HRP-conjugated goat polyclonal anti-rabbit (111-035-003, Jackson ImmunoResearch); and HRP-conjugated goat polyclonal anti-mouse (115-035-003, Jackson ImmunoResearch).

### Immunoprecipitation (IP)

Primary neurons and HEK293T cells were harvested and lysed as described above. The cell lysates were then subjected to IP with the indicated antibodies conjugated with magnetic beads using the Pierce™ Crosslink Magnetic IP/Co-IP kits (88805, Thermo Fisher). The immunoprecipitates were washed multiple times with the lysis buffer and analyzed using immunoblotting.

For the *in vivo* ubiquitination assay, denatured protein IP was conducted. Initially, cells were lysed using a low-salt lysis buffer, and the resulting supernatants were treated at 95°C for 5 minutes in the presence of 1% SDS to denature the proteins. Subsequently, the denatured cell lysates underwent dilution with lysis buffer to reduce the SDS concentration to below 0.1%. Following centrifugation, IP was performed, and the samples were analyzed via immunoblotting.

### Statistical analysis

All experiments were performed independently at least three times and data were represented as mean ± SEM. Statistical analysis was carried out using Prism v8.0 (GraphPad Software). Two-tailed unpaired Student's *t* test was used for comparisons between two experimental groups. One- or two-way analysis of variance (ANOVA) followed by post-hoc Bonferroni correction was used to compare more than two groups. Pearson's correlation coefficient was used in correlation analysis. P < 0.05 was considered significant.

## Results

### TRIM32 promotes neuronal ferroptosis *in vitro*

**Figure 1:**
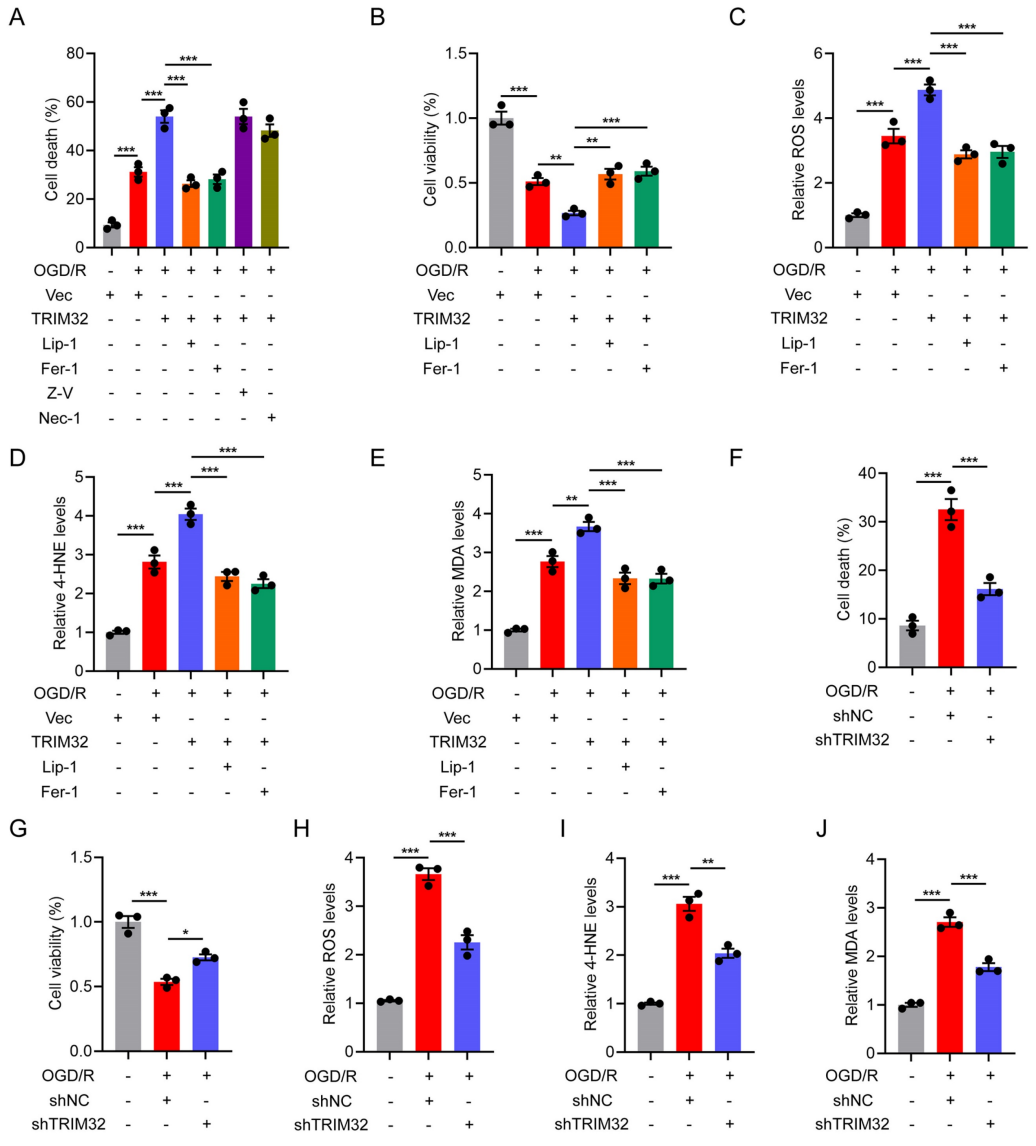
** TRIM32 promotes neuronal ferroptosis *in vitro.*** (A) Cell death was assessed using Live/Dead staining and the PI positive primary neurons were quantified in vector or TRIM32-overexpression groups exposed to OGD/R for 2 hours in combination with the indicated inhibitors. (B-E) Cell viability (B), ROS (C), 4-HNE (D) and MDA (E) levels were assessed in vector or TRIM32-overexpression groups exposed to OGD/R for 2 hours in combination with Lip-1 or Fer-1. (F) Cell death was assessed using Live/Dead staining and the PI positive primary neurons were quantified in shNC or shTRIM32 groups exposed to OGD/R for 2 hours. (G-J) Cell viability (G), ROS (H), 4-HNE (I) and MDA (J) levels were assessed in shNC or shTRIM32 groups exposed to OGD/R for 2 hours. Data were evaluated using one-way ANOVA followed by post-hoc Bonferroni correction (A-J).

We firstly wondered if TRIM32 exert beneficial or detrimental effects on primary neurons upon oxygen glucose deprivation and reperfusion (OGD/R) injury *in vitro*. OGD/R significantly inhibits neuronal cell viability and promotes cell death (Figs. 1A and 1B). Interestingly, we found that overexpression of TRIM32 aggravated the downregulated cell viability and promoted cell death while downregulation of TRIM32 reversed these detrimental effects (Figs. 1A and 1B, 1F and 1G). As cell death was promoted in neurons upon TRIM32 overexpression, we further wondered which cell death pathway including apoptosis, ferroptosis and necrosis involved in this process. As shown in Fig. 1A, Liproxstatin-1 (Lip-1, 1 μM) and Ferrostatin-1 (Fer-1, 10 μM), which are ferroptosis inhibitors could markedly reverse the promoted cell death upon TRIM32 overexpression, while Z-VAD-FMK (an apoptosis inhibitor, 100 μM) or Necrostatin-1 (a necrosis inhibitor, 100 μM) showed little effect, indicating that TRIM32 may promote neuronal cell death through ferroptosis upon OGD/R *in vitro*. In keeping with this finding, we found that TRIM32 overexpression increased ROS and lipid peroxidation levels (Figs. 1C-1E), both of which are important biological processes in ferroptosis, while silence of TRIM32 downregulated ROS and lipid peroxidation levels in neurons upon OGD/R (Figs. 1H-1J). Consistent with above results, we also demonstrated that TRIM32 promoted neuronal ferroptosis upon treating with a classic ferroptosis activator Erastin in neurons (Fig. S1). In all, these results showed that TRIM32 promotes neuronal ferroptosis *in vitro*.

### Neuron-specific silence of *Trim32* inhibited neuronal ferroptosis and promoted locomotor functional recovery after SCI

**Figure 2:**
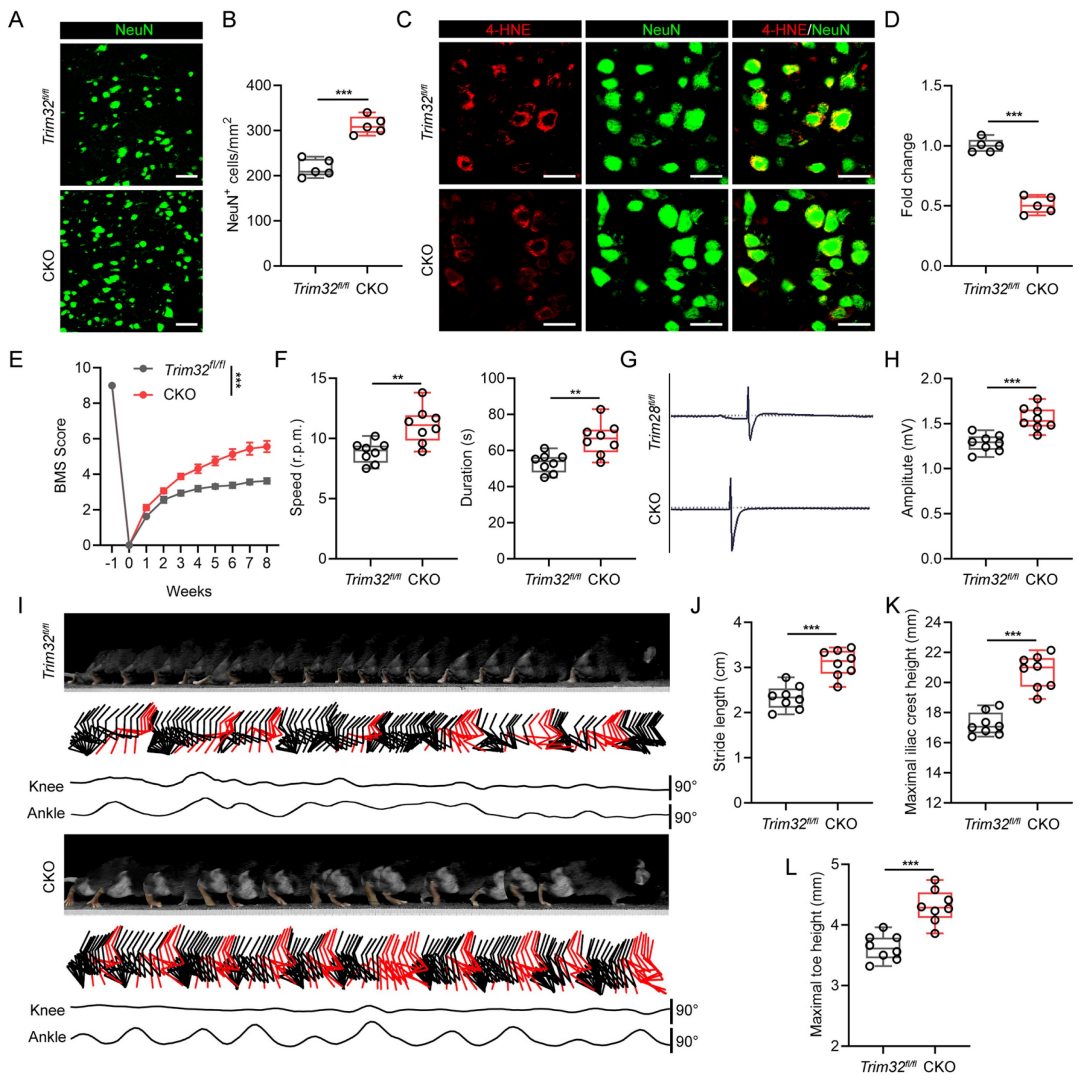
** Neuron-specific silence of *Trim32* inhibited neuronal ferroptosis and promoted locomotor functional recovery after SCI.** (A-B) Representative images and quantification of NeuN^+^ neurons from *Trim32^fl/fl^* and *Trim32* CKO mice at 8 wpi. Scale bar = 100 μm. (C-D) Representative images and quantification of NeuN and 4-HNE co-immunostaining at 1 dpi. Scale bar = 100 μm. (E) BMS score in *Trim32^fl/fl^* and *Trim32* CKO mice at the indicated time after SCI (n=8 per group). (F) Rotarod tests in mice at 8 wpi. (G-H) Representative images and quantification of EMG recordings of gastrocnemius muscle in mice at 8 wpi. (I) Chronophotograph, the corresponding color-coded stick view decomposition of hindlimb movements, and knee and ankle angle oscillation trace in mice at 8 wpi. (J-L) Stride length(J), maximal iliac crest (K), and toe height (L) in mice at 8 wpi. Data were evaluated using two-tailed unpaired Student's *t*-test (B, D, F, H and J-L) and two-way ANOVA followed by post-hoc Bonferroni correction (E).

As there are several cell types in the spinal cord after SCI, we further determined which cell type TRIM32 mainly located in. As shown in Fig. S2, TRIM32 was co-immunostained with NeuN (neuron maker), GFAP (astrocyte marker), IBA (microglia/macrophage marker) and CD31 (endothelial cell marker). It was found that TRIM32 was mainly expressed in neurons in spinal cord after SCI (Fig. S2). To further determine the role of TRIM32 in neuronal ferroptosis *in vivo*, neuron-specific conditional knockout of *Trim32* (*Trim32* CKO) were generated by crossing *Trim32^fl/fl^* mice with mouse lines expressing Cre recombinase under control of neuron-specific Synapsin I promotor^24^. We performed a severe spinal cord contusion injury model which allows spontaneous functional recovery and replicates the most common human SCI pathophysiology^21^, ^22^. We firstly checked the number and status of neurons adjacent to the lesion core. Immunofluorescence analysis of NeuN in representative area 1,000 μm adjacent to the lesion core showed that ablation of *Trim32* markedly prevented neuronal loss (Figs. 2A and 2B). Additionally, the intensity of 4-HNE, which is a lipid peroxidation marker in neuronal cells was significantly inhibited in *Trim32* CKO mice (Figs. 2C and 2D). Next, a series of functional experiments were used to evaluate the role of neuronal TRIM32 in neurological function. Knockout of neuronal *Trim32* was shown to promote hindlimb motor function according to the Basso mouse scale (BMS) score and rotarod test (Figs. 2E and 2F)^25^, ^26^. The hindlimb muscle electromyography (EMG) recording results showed that *Trim32* CKO led to improved motor-evoked potential (MEP) signal after SCI (Figs. 2G and 2H). Hindlimb kinematic analysis showed that depletion of neuronal *Trim32* exhibited frequent plantar stepping and increased angle oscillation of hindlimb joints 8 weeks post-injury (wpi) (Figs. 2I-2L). These results indicated that depletion of neuronal *Trim32* could inhibit neuronal ferroptosis and promote locomotor functional recovery in mice after SCI.

### Neuron-specific overexpression of *Trim32* promoted neuronal ferroptosis and impaired locomotor functional recovery, which could be reversed by Lip-1 after SCI

**Figure 3:**
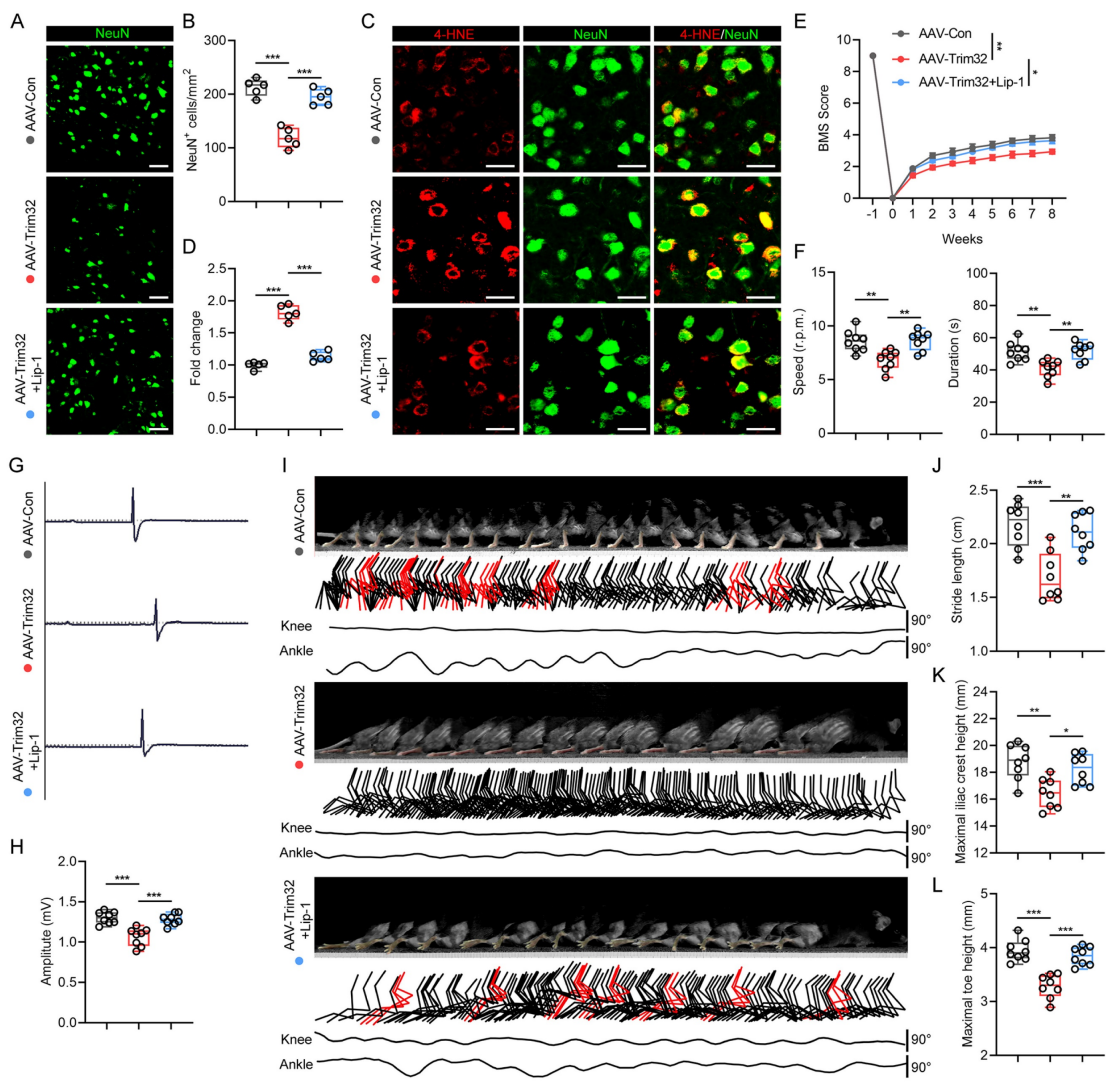
** Neuron-specific overexpression of *Trim32* promoted neuronal ferroptosis and impaired locomotor functional recovery, which could be reversed by Lip-1 after SCI.** (A-B) Representative images and quantification of NeuN^+^ neurons in AAV-Con, AAV-Trim32, and AAV-Trim32+Lip-1 mice at 8 wpi. Scale bar = 100 μm. (C-D) Representative images and quantification of NeuN and 4-HNE co-immunostaining at 1 dpi. Scale bar = 100 μm. (E) BMS scores for AAV-Con, AAV-Trim32, and AAV-Trim32+Lip-1 mice at indicated times after SCI (n = 8 per group). (F) Rotarod tests in mice at 8 wpi. (G-H) Representative images and quantification of EMG recordings of gastrocnemius muscle in mice at 8 wpi. (I) Chronophotograph, the corresponding color-coded stick view decomposition of hindlimb movements, and knee and ankle angle oscillation trace in mice at 8 wpi. (J-L) Stride length(J), maximal iliac crest (K), and toe height (L) in mice at 8 wpi. Data were evaluated using one-way ANOVA followed by post-hoc Bonferroni correction (B, D, F, H, and J-L) and two-way ANOVA followed by post-hoc Bonferroni correction (E).

To further verify the role of neuronal TRIM32 in promoting ferroptosis after SCI, we construct neuron-specific adeno-associated virus (AAV-PHP.eB-hSyn) to specifically overexpress *Trim32* in neurons *in vivo*. Western blotting and immunofluorescence staining confirmed the specific overexpression of AAV-Trim32 in spinal cord neurons (Fig. S3). Immunofluorescence staining showed that overexpression of *Trim32* induced a greater neuronal loss and 4-HNE level in mice after SCI (Figs. 3A-3D). Also, the BMS score, rotarod test, EMG recording, and hindlimb kinematic analysis indicated that TRIM32 overexpression resulted in a limited locomotor functional recovery compared with AAV-Con group (Figs. 3E-3L). However, these detrimental effects when overexpressing *Trim32* were markedly reversed when treating ferroptosis inhibitor Lip-1 *in vivo* (Figs. 3A-3L). Taken together, these results showed that *Trim32* overexpression aggravated neuronal ferroptosis and inhibited locomotor functional recovery in mice after SCI.

### TRIM32 interacts with and promotes autophagic degradation of GPX4

**Figure 4:**
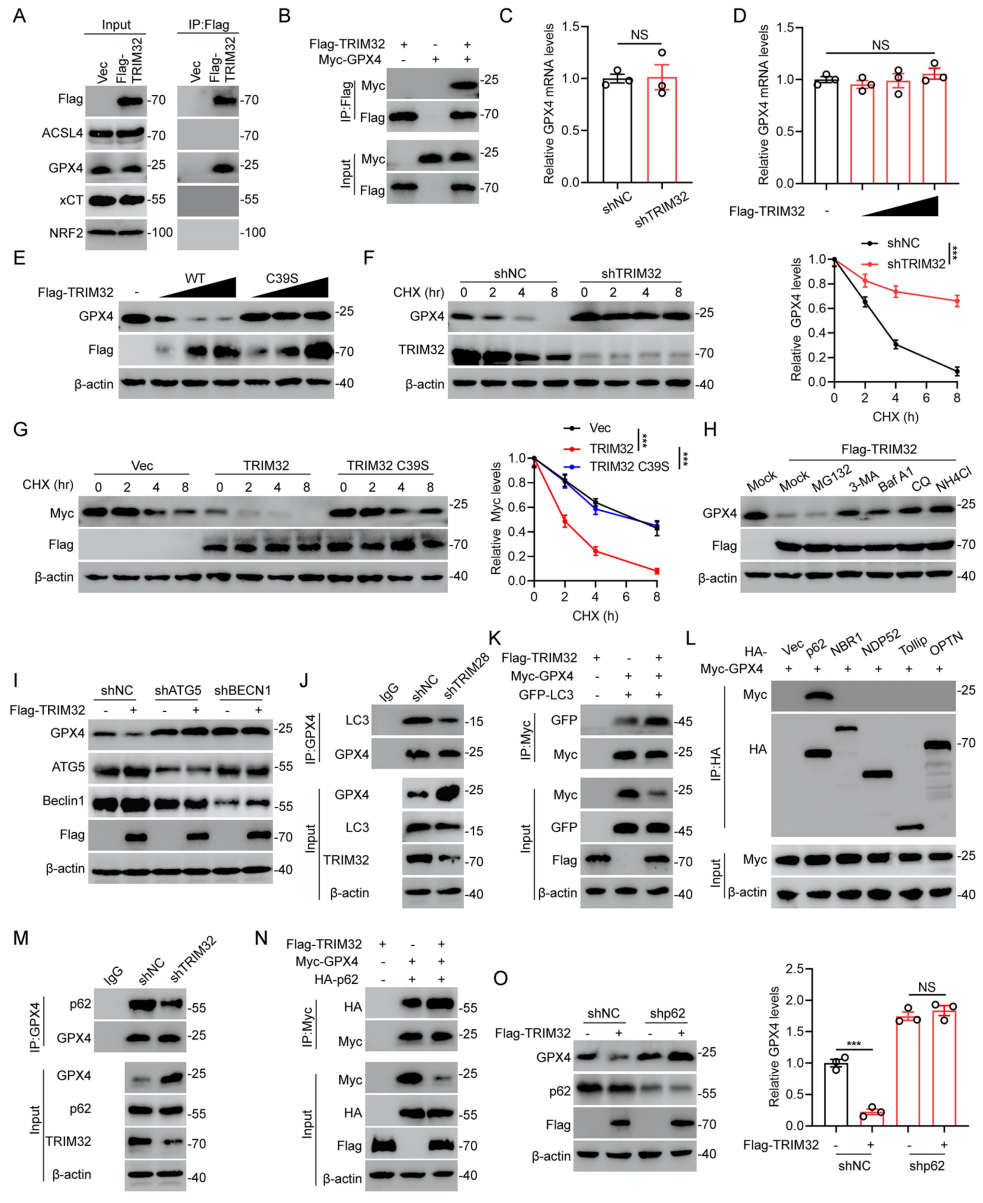
** TRIM32 interacts with and promotes autophagic degradation of GPX4.** (A) Primary neurons transfected with Flag-TRIM32 were immunoprecipitated with anti-Flag and immunoblotted with Flag, ACSL4, GPX4, xCT and NRF2 in the presence of Baf A1. (B) Co-IP assays were carried out to investigate the interaction between exogenous TRIM32 and GPX4 in HEK293T cells in the presence of Baf A1. (C-D) GPX4 mRNA levels in indicated groups. (E) Increasing amounts of Flag-tagged TRIM32 (WT or C39S mutant) were transfected into primary neurons and cell lysates were analyzed using immunoblotting with anti-GPX4 and anti-Flag. (F-G) Indicated protein levels in primary neurons (F) or HEK293T cells (G) in the absence or presence of CHX (10 μg/mL) for indicated time periods. (H) GPX4 protein levels in primary neurons transfected with Flag-TRIM32 and treated with DMSO (Mock), MG132, 3-MA, Baf A1, CQ, or NH4CL. (I) Primary neurons transfected with scramble shRNA or ATG5- or Beclin 1-specific shRNAs were co-transfected with vector or Flag-TRIM32 and lysates were analyzed using immunoblotting. (J) Co-IP analysis of endogenous GPX4 and LC3 with or without TRIM32 knockdown in primary neurons. (K) Co-IP analysis of exogenous GPX4 and LC3 in HEK293T cells with or without TRIM32 overexpression. (L) Co-IP analysis of GPX4 and cargo receptors in HEK293T cells in the presence of Baf A1. (M) Co-IP analysis of endogenous GPX4 and p62 in primary neurons with or without TRIM32 knockdown. (N) Co-IP analysis of exogenous GPX4 and p62 with or without TRIM32 overexpression in HEK293T cells. (O) Primary neurons transfected with scramble shRNA or p62-specific shRNAs were co-transfected with vector or Flag-TRIM32 and lysates were analyzed by immunoblotting. Data were evaluated using two-tailed unpaired Student's *t*-test (C, O), one-way ANOVA followed by post-hoc Bonferroni correction (D) and two-way ANOVA followed by post-hoc Bonferroni correction (F-G).

To further investigate the underlying mechanism by which TRIM32 promotes neuronal ferroptosis, we examined the potential protein-protein interaction between TRIM32 and several key ferroptosis-related proteins including ACSL4, GPX4, xCT and NRF2. As shown in Fig. 4A, we found that only endogenous GPX4 could interacted with ectopically expressed Flag-TRIM32 in primary neurons. The interaction between Flag-TRIM32 and Myc-GPX4 was also exogenously verified in HEK293T cells (Fig. 4B). These data indicated that TRIM32 specifically interacted with GPX4.

Since TRIM32 is an E3 ubiquitin ligase and was reported to mediate the protein stability, we further wondered if TRIM32 exert a crucial role in GPX4 stability. At first, we found that the mRNA levels of GPX4 was not altered when overexpressing or silencing TRIM32 (Figs. 4C and 4D). Interestingly, increasing amounts of TRIM32 decreased the protein level of endogenous GPX4 while a catalytically inactive TRIM32 mutant with C39S did not (Fig. 4E). In the presence of protein synthesis inhibitor cycloheximide (CHX), silence of TRIM32 significantly promoted the stability of GPX4 in primary neurons (Fig. 4F). In contrast, overexpression of TRIM32 markedly enhanced the protein degradation of GPX4, while TRIM32 C39S did not in the presence of CHX (Fig. 4G). These results indicated that TRIM32 regulated GPX4 expression at the translational but not the transcriptional level.

The ubiquitin-proteasome and autophagy-lysosome pathways are widely recognized as the two primary mechanisms for mediating protein degradation. To figure out which degradation system be involved in TRIM32-mediated GPX4 degradation, we used proteasome inhibitor MG132 and autophagy and lysosome inhibitor including 3-methyladenine (3-MA), bafilomycin A1 (Baf A1), chloroquine (CQ), and NH_4_CI to treat primary neurons with TRIM32 overexpression. Expectedly, overexpression of TRIM32 lead to GPX4 protein degradation, which could be inhibited by 3-MA, Baf A1, CQ and NH_4_CI but not MG132, indicating that TRIM32 overexpression-induced GPX4 protein degradation was mainly through autophagy-lysosome pathway (Figs. 4H and S4A). ATG5 and Beclin 1 are two key regulators for autophagy and their silence could result in impaired autophagy. GPX4 degradation mediated by TRIM32 overexpression was significantly inhibited when ATG5 or Beclin 1 depletion in primary neurons, further suggesting the TRIM32-induced GPX4 degradation was primary depend on autophagy (Figs. 4I and S4B). In addition, GPX4 degradation was promoted when treatment of Rapamycin and EBSS, both of which are classic autophagy inducers (Figs. S4C and S4D). Moreover, TRIM32 depletion-induced GPX4 stability was abolished after Rapamycin treatment, while Rapamycin or EBSS treatment induced GPX4 degradation was reversed after Baf A1 treatment (Figs. S4E and S4F). Depletion of TRIM32 inhibited the interaction between GPX4 and LC3, which is an autophagosomes marker while overexpression of TRIM32 promoted the association (Figs. 4J and 4K). In all, these data further demonstrated that TRIM32 promoted GPX4 protein degradation was mainly depended on the autophagy-lysosome pathway.

Increasing evidence showed that cargo receptors play critical roles in transmitting substrates for selective autophagic degradation^27^. Accordingly, we found that GPX4 specifically interacted with the cargo receptor p62 (Fig. 4L). Interestingly, the association between GPX4 and p62 was upregulated when overexpressing TRIM32 while downregulated when silencing TRIM32 (Figs. 4M and 4N). In addition, knockdown of p62 markedly inhibited the GPX4 degradation when overexpressing TRIM32 (Fig. 4O), further indicating that TRIM32 promoted GPX4 autophagic degradation through the cargo receptor p62.

### TRIM32 promoted K63-linked ubiquitination of GPX4

**Figure 5:**
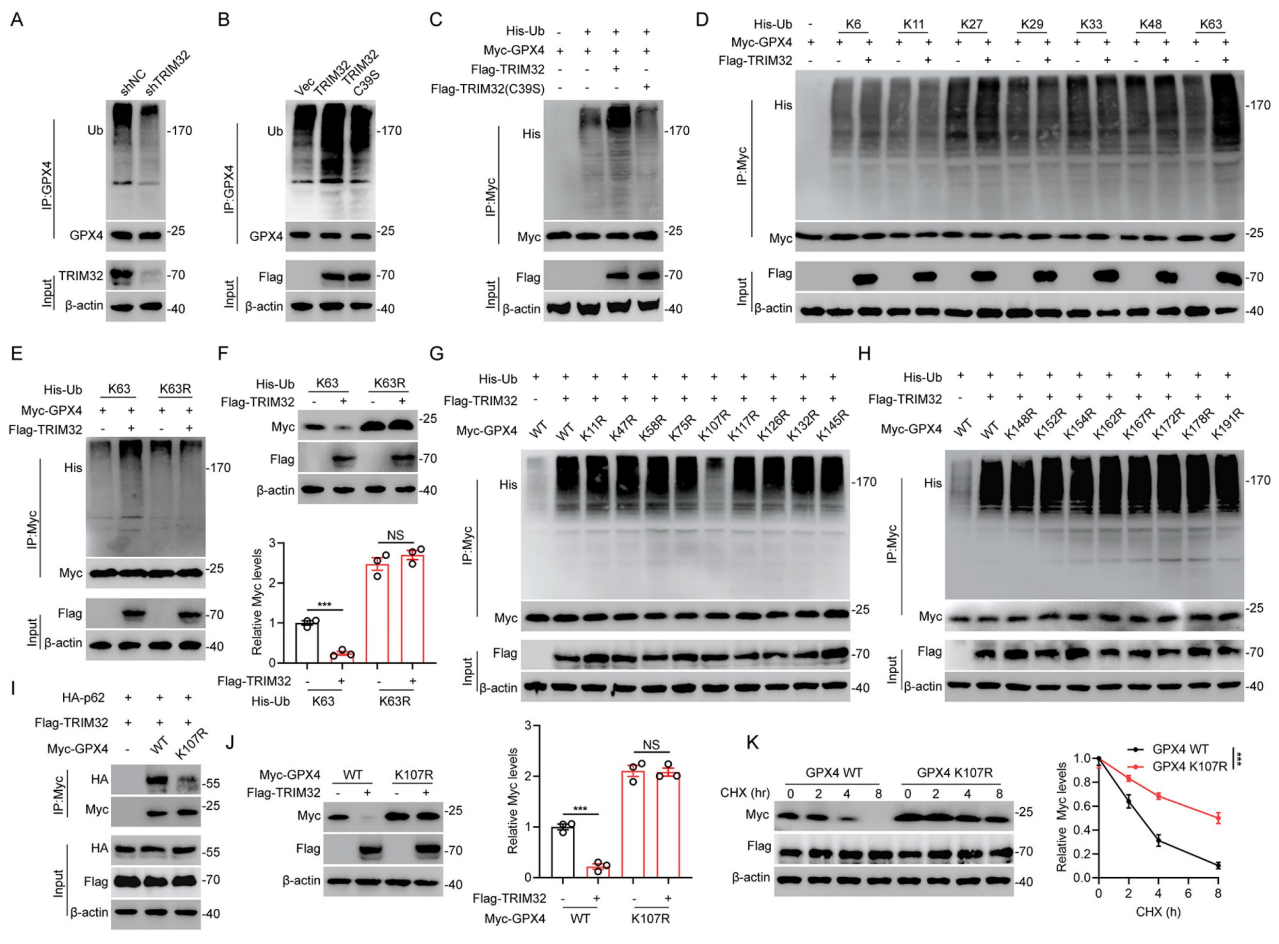
** TRIM32 promoted K63-linked ubiquitination of GPX4.** (A-B) Lysates from primary neurons transfected with (A) shNC or shTRIM32 and (B) Flag-tagged TRIM32 (WT or C39S) were immunoprecipitated with anti-GPX4 and examined with anti-ubiquitin and anti-GPX4. (C) Lysates from HEK293T cells co-transfected with His-Ub and Myc-tagged GPX4 together with Flag-tagged TRIM32 (WT or C39S) were immunoprecipitated with anti-Myc followed by immunoblotting with anti-His and anti-Myc. (D) HEK293T cells were co-transfected with Flag-TRIM32, Myc-GPX4, and the indicated His-Ub plasmids and Myc-GPX4 ubiquitylation linkage was examined. (E) Co-IP analysis of HEK293T cells expressing Myc-GPX4 and His-ubiquitin (K63 or K63R) in the absence or presence of Flag-TRIM32. (F) Lysates of HEK293T cells transfected with K63 or K63R Ub in the presence of Flag-TRIM32 and Myc-GPX4 were analyzed and quantified by immunoblotting. (G-H) Co-IP analysis of HEK293T cells transfected with His-Ub, Flag-TRIM32 and Myc-GPX4 or indicated mutants. (I) Co-IP analysis of interaction between HA-p62 and Myc-GPX4 (WT or K107R) in HEK293T cells. (J) Immunoblot analysis and quantification of HEK293T cell extracts transfected with Flag-TRIM32 and Myc-GPX4 (WT or K107R). (K) Lysates from HEK293T cells co-transfected with Myc-tagged GPX4 (WT or K107R) with Flag-TRIM32 were immunoblotted with anti-Flag and anti-Myc in the absence or presence of CHX (10 μg/mL) for indicated time periods. Data were evaluated using two-tailed unpaired Student's *t*-test (F, J) and two-way ANOVA followed by post-hoc Bonferroni correction (K).

Since TRIM32 is an E3 ubiquitin ligase and ubiquitination is related to protein stabilization, we further wondered whether TRIM32 regulated GPX4 ubiquitination and further degradation. Depletion of TRIM32 inhibited while overexpression of TRIM32 promoted GPX4 ubiquitination in primary neurons and HEK293T cells (Figs. 5A-5C). Moreover, TRIM32 C39S mutant exert no effect on endogenous or exogenous GPX4 ubiquitination in primary neurons and HEK293T cells (Figs. 5B and 5C). To further determine which type of ubiquitin chain was response for TRIM32-mediated GPX4 ubiquitination, we transfected HEK293T cells with TRIM32, GPX4 and different forms of ubiquitin chain including K6-, K11-, K27-, K29-, K33-, K48-, or K63-linked chains. As shown in Fig. 5D, TRIM32 significant enhanced K63-linked ubiquitin chain level of GPX4. Additionally, the downregulated protein and upregulated ubiquitination level of GPX4 after TRIM32 overexpression were significantly abolished when transfecting K63R ubiquitin mutants (Figs. 5E and 5F). To further determine the exact ubiquitination site response for TRIM32-induced GPX4 ubiquitination, we constructed 17 GPX4 mutants with lysine (K) to arginine (R) substitution and co-transfected each GPX4 mutant into HEK293T cells together with Flag-TRIM32 and His-K63 Ub. As shown in Figs. 5G and 5H, only K107R mutant significantly abolished the ubiquitination level. In addition, K107R mutant significantly reduced the interaction between GPX4 and p62 (Fig. 5I). Accordingly, overexpression of TRIM32 induced a decrease in the WT but not the K107R GPX4 protein level (Fig. 5J). Moreover, TRIM32 was unable to lead to K107R GPX4 protein degradation the presence of CHX (Fig. 5K). Collectively, these results demonstrated that TRIM32 promoted K63-linked ubiquitination of GPX4 at K107.

### TRIM32 promoted neuronal ferroptosis by destabilizing GPX4 *in vitro*

Since TRIM32 promoted GPX4 degradation, we next investigated the role of TRIM32/GPX4 axis in neuronal ferroptosis. The promoted neuronal cell death, downregulated cell viability, upregulated ROS and lipid peroxidation upon TRIM32 overexpression were largely reversed by overexpressing GPX4 upon OGD/R in primary neurons (Fig. S5). These data demonstrated the TRIM32 promoted neuronal ferroptosis through a GPX4-dependent mechanism in primary neurons *in vitro*.

### ROS-ATM-Chk2 signaling pathway phosphorylates TRIM32 and forms a positive feedback loop

**Figure 6:**
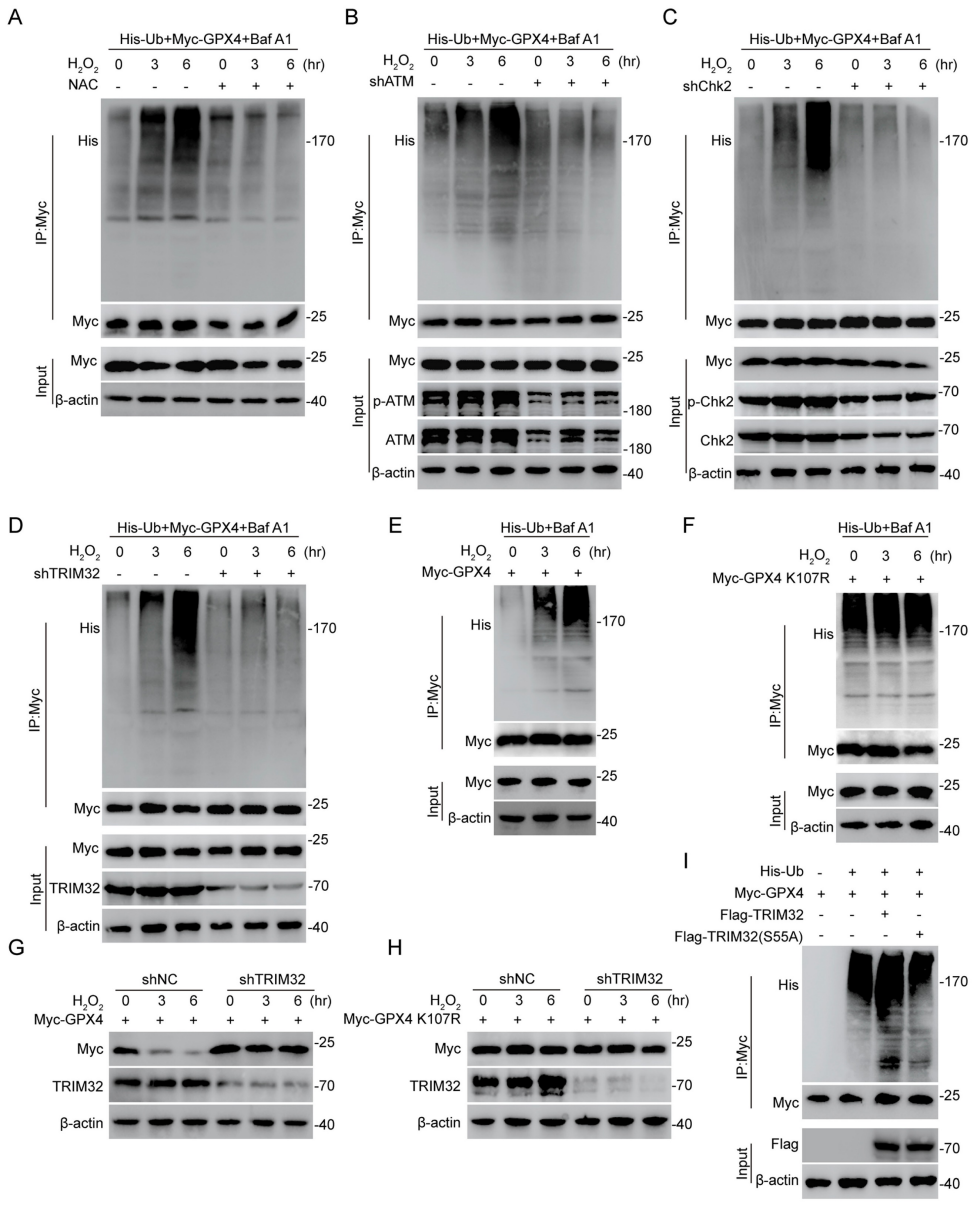
** ROS-ATM-Chk2 signaling pathway phosphorylates TRIM32 and forms a positive feedback loop.** (A) Lysates from HEK293T cells co-transfected with Myc-GPX and His-Ub were immunoprecipitated with anti-Myc and then immunoblotted with anti-Myc and anti-His with 0.1 mM H_2_O_2_ treatment for 0 to 6 hours in the absence or presence of NAC (3nM). (B-D) Lysates from HEK293T cells co-transfected with Myc-GPX, His-Ub and shATM (B) or shChk2 (C) or shTRIM32 (D) were immunoprecipitated with anti-Myc and then immunoblotted with anti-Myc and anti-His with 0.1 mM H_2_O_2_ treatment for 0 to 6 hours. (E-F) Lysates from HEK293T cells co-transfected with Myc-GPX4 WT (E) or K107R (F) and His-Ub were immunoprecipitated with anti-Myc and then immunoblotted with anti-Myc and anti-His with 0.1 mM H_2_O_2_ treatment for 0 to 6 hours. (G-H) Lysates from HEK293T cells co-transfected with Myc-GPX4 WT (G) or K107R (H) in the absence or presence of shTRIM32 were analyzed by immunoblotting with anti-Myc and anti-TRIM32 with 0.1 mM H_2_O_2_ treatment for 0 to 6 hours. (I) Lysates from HEK293T cells co-transfected with His-Ub and Myc-tagged GPX4 together with Flag-tagged TRIM32 (WT or S55A) were immunoprecipitated with anti-Myc followed by immunoblotting with anti-His and anti-Myc.

A prominent feature of spinal cord microenvironment after SCI is under high ROS^4^, ^28^. Also, promoted ferroptosis could lead to increasing lipid ROS levels. Interestingly, a recent study showed the ROS-ATM-Chk2-TRIM32 signaling pathway under metabolic stress^29^. Then, we wondered if this mechanism occurs in TRIM32-mediated GPX4-dependent neuronal ferroptosis. We found that the ubiquitination level of GPX4 was increased over time under 0.1 mM H_2_O_2_ for different time periods, while unchanged upon N-acetylcysteine (NAC), a ROS scavenger treatment in primary neurons (Fig. 6A). To further confirm the ATM-Chk2-TRIM32 axis in neuronal ferroptosis, we silenced ATM, Chk2 and TRIM32 respectively and found that increasing ubiquitination level of GPX4 upon H_2_O_2_ treatment was abolished in shATM, shChk2 or shTRIM32 primary neurons (Figs. 6B-6D), further indicated the ROS-ATM-Chk2-TRIM32 signaling pathway to promote GPX4 ubiquitination. In addition, the ubiquitination level of GPX4 was unchanged when transfecting with K107R GPX4 compared with WT GPX4 even in the presence of H_2_O_2_ (Figs. 6E and 6F). To further elucidate the impact of oxidative stress on TRIM32-medicated GPX4 degradation, we silenced endogenous TRIM32 and found that depletion of TRIM32 reversed the degradation of GPX4 upon H_2_O_2_ treatment (Fig. 6G). However, depletion of TRIM32 had no effect on GPX4 K107R mutants (Fig. 6H). In all, these data showed a ROS-ATM-Chk2-TRIM32 signaling pathway in promoting GPX4 ubiquitination and degradation, suggesting a positive feedback loop between ROS and TRIM32.

Increasing reports showed that the recognition and activity of TRIM family proteins were dependent on its phosphorylation status^30^, ^31^. Interestingly, TRIM32 was reported to be phosphorylated by Chk2 at the S55 site and its E3 ubiquitin ligase activity was dependent on this phosphorylation^29^. To further prove this, we first treated HEK293T cells with λ-phosphatase to dephosphorylate and found that the interaction between TRIM32 and GPX4 was markedly suppressed (Fig. S6). Moreover, the increased GPX4 ubiquitination level upon WT TRIM32 overexpression was abolished when transfecting with TRIM32 S55A mutant, indicating that the phosphorylation status at S55 site is crucial for its E3 ubiquitin ligase activity (Fig. 6I). Taken together, these results indicated the ROS-ATM-Chk2 signaling pathway phosphorylates TRIM32 at S55, contributing to GPX4 ubiquitination and degradation.

### Clinical high serum and cerebrospinal fluid (CSF) lipid peroxidation levels positively correlated with poor neurological function in patients with SCI

To investigate whether lipid peroxidation levels correlated with neurological function in patients with SCI, we recruited different groups of patients with different American Spinal Injury Association Impairment Scale (ASIA) grades which indicates SCI severity after SCI and examining serum MDA levels individually (Fig. S7A). As shown in Fig. S7B, MDA levels were significantly increased in patients with SCI compared with the control group. Moreover, MDA levels were increased with the SCI severity (Fig. S7C). Increasing evidence showed a dysregulated CSF microenvironment in some CNS disorders^32^, ^33^. Then, we collected CSF from patients with SCI and control subjects and found that MDA levels in CSF were markedly elevated in patients with SCI and indicated poor neurological function (Figs. S7D-S7F). The hyperintensity area in injured spinal cord on magnetic resonance imaging (MRI) T2-weighted images is related to the destruction of the spinal cord and indicates poor neurological function after injury. The MDA levels in CSF of patients with SCI were positively correlated with the hyperintensity area in injured spinal cord (Figs. S7G and S7H). Taken together, these clinical results indicated a positive correlation between promoted lipid peroxidation in serum and CSF and poor neurological function in patients with SCI.

## Discussion

**Figure 7:**
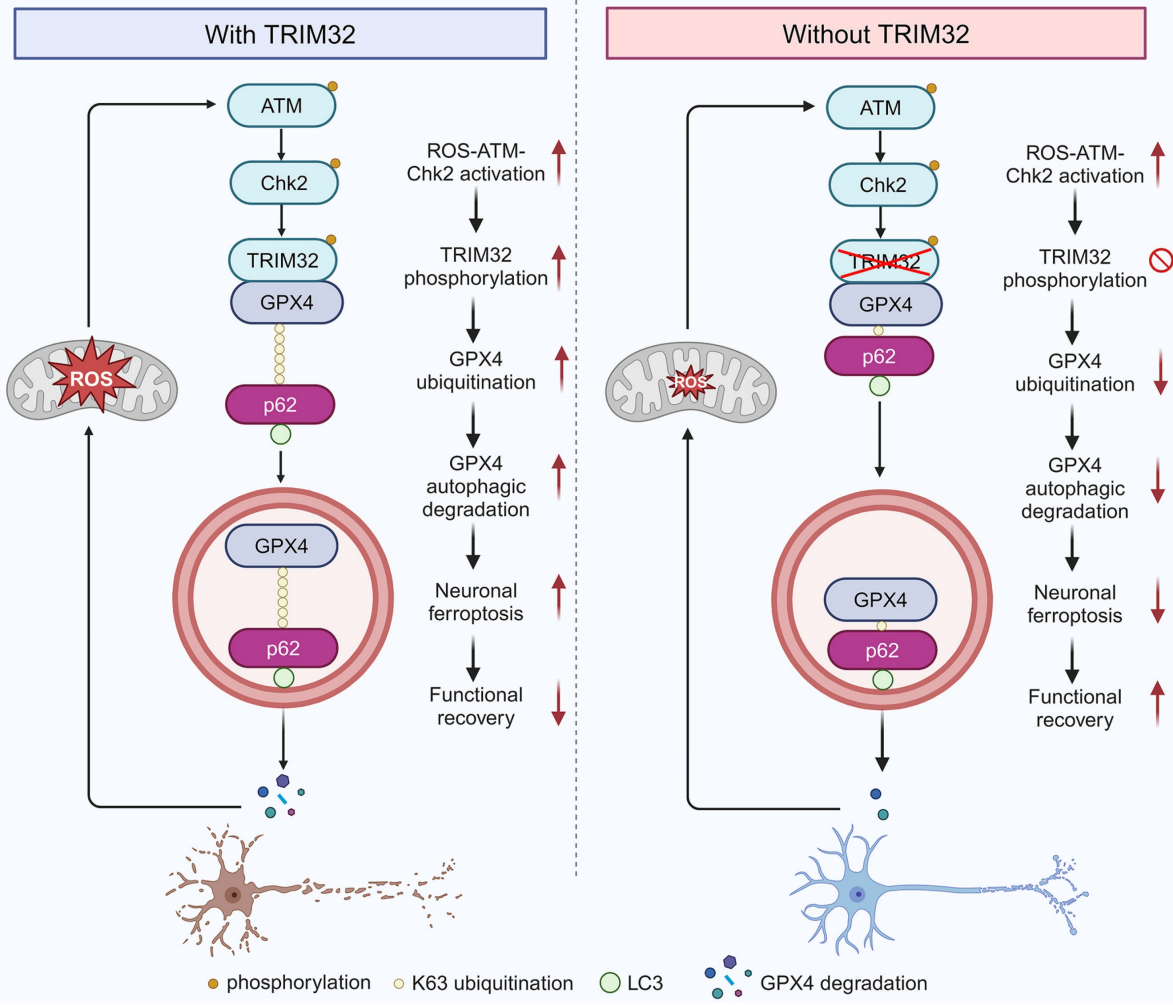
** Proposed mechanism underlying TRIM32 in enhancing neuronal ferroptosis after SCI.** TRIM32 promotes K63-linked ubiquitination of GPX4 at K107, thereby enhancing p62-GPX4 interaction and leading to p62-dependent selective autophagic degradation of GPX4. ROS-ATM-Chk2 signaling pathway phosphorylates TRIM32 at S55, leading to GPX4 degradation and subsequent neuronal ferroptosis after SCI, suggesting a positive feedback loop between ROS and TRIM32. In the absence of TRIM32, GPX4 protein is stabilized and neuronal ferroptosis is inhibited, leading to better locomotor functional recovery after SCI.

This present study identified E3 ubiquitin ligase TRIM32 as a crucial regulator for neuronal ferroptosis after SCI. TRIM32 catalyzed the conjugation of K63-linked ubiquitin to GPX4 at K107, which is key for p62-mediated selective autophagic degradation, further contributing to GPX4 degradation and enhancing neuronal ferroptosis. After SCI, activation of ROS-ATM-Chk2 signaling pathway phosphorylated TRIM32 at S55 and further contributed to GPX4 degradation and neuronal ferroptosis. Genetic depletion of *Trim32* in neurons precluded neuronal ferroptosis and neuronal loss, and promoted locomotor functional recovery in mice after SCI (Fig. 7).

ROS accumulation is a key feature of secondary SCI. It is well-established that ROS can induce various forms of cell death, including apoptosis, autophagy, and ferroptosis. Maintaining a proper balance of ROS is crucial for neuronal survival and axonal regeneration. While studies have predominantly focused on therapeutic strategies to reduce ROS production, the roles of ROS in underlying mechanisms have been less explored. This study uncovers a novel role for ROS in activating the TRIM32/GPX4 axis and suggests the existence of a positive feedback loop between ROS and TRIM32, which may enhance our understanding of ROS dynamics following CNS injury.

GPX4, one of the most important antioxidant enzymes, could protect cells from ferroptosis for its role in converting lipid hydroperoxides into non-toxic lipid alcohols and is recognized as an important inhibitor of ferroptosis^34^, ^35^. Thus, genetic overexpression or pharmacological activation may thus be a promising approach for desensitizing cells to ferroptosis^36^. The transcription factor NRF2 could regulate GPX4 transcriptional level^37^, ^38^. Recently, various post-translational modifications of GPX4 have been reported^39^, ^40^. For example, Guo *et al.* reported that TRIB2 could stabilize GPX4 and attenuate oxidative stress-induced cell damage^41^. Another study showed that CST1 inhibited ferroptosis by recruiting OTUB1 and subsequent reducing GPX4 ubiquitination and degradation^42^. A recent study showed that CKB could bind to and phosphorylate GPX4 at S104, thereby inhibiting chaperone-mediated autophagic degradation of GPX4 by HSC70 and promoting tumorigenesis^43^. However, the detailed ubiquitination site and type of GPX4 and the regulatory mechanism remains undetermined, especially in the context of SCI. It was speculated that GPX4 could be ubiquitinated at K107, K162 and K167 according to previous proteomic datasheets^44^, ^45^. Interestingly, our results indicated that TRIM32 ubiquitinated GPX4 at K107, which further confirmed the proteomic analysis. This study uncovered the interaction between GPX4 ubiquitination and selective autophagic degradation during neuronal ferroptosis. TRIM32 catalyzed K63-linked ubiquitination of GPX4 at K107, thereby promoting p62-mediated autophagic degradation and destabilization of GPX4, providing a novel insight into the post-translational modification of GPX4. Since TRIM32 is an E3 ubiquitin ligase, it's interesting to explore whether other deubiquitinase could cleave K63-linked ubiquitination of GPX4 and counteract TRIM32-mediated GPX4 ubiquitination and neuronal ferroptosis in future. Moreover, whether GPX4 ubiquitination have synergistic or antagonistic effects with other post-translational modifications including phosphorylation, SUMOlyation, methylation, *et al.* need investigations.

Accumulating evidence underscores the critical role of substrate ubiquitination in selective autophagy^46^, ^47^. Autophagic cargo receptors selectively bind to cargo substrates, facilitating their delivery to autophagosomes for subsequent degradation. Zhi *et al.* reported that RBX1 could ubiquitinate p85α at K256, which could be recognized by the autophagy cargo receptor OPTN, leading its autophagic degradation^48^. NEDD4 has been shown to catalyze K27-linked polyubiquitination of TBK1 at K344, thereby facilitating NDP52-mediated selective autophagic degradation^49^. Another study revealed that K48-linked ubiquitination of ACE2 at K187 enhanced TOLLIP-dependent autophagic degradation^50^. In addition, it was reported that TRIM11 dampens Treg cell differentiation by enhancing the ubiquitination and selective autophagic degradation of AIM2 in a p62-dependent manner^51^. In the present investigation, it was noted that the functions of TRIM32 were compromised in p62-depleted cells. Moreover, it was demonstrated that the cargo receptor p62 recruited and delivered GPX4 to autophagosomes for subsequent degradation. Furthermore, p62 induced the autophagic degradation of K63-linked ubiquitylated GPX4. These findings unveil a novel mechanism underlying TRIM32-mediated autophagic degradation of GPX4 in a p62-dependent manner, characterized by dynamic interplay between ubiquitination and selective autophagic degradation, and offer a potential therapeutic target for SCI treatment.

The present study encountered several limitations. Firstly, due to restricted access to human spinal cord samples, serum and CSF were utilized to assess lipid peroxidation in patients with SCI, which may not fully reflect the local injured spinal cord microenvironment. Moreover, including ASIA rating scale data would enhance the robustness and validity of our findings. Second, whether other E3 ubiquitin ligases might be involved in neuronal ferroptosis after SCI require further studies. Third, further studies are encouraged to develop TRIM32-specific antagonists or drugs which could block the interaction between TRIM32 and GPX4 for potential clinical applicability. Also, ferroptosis is prevalent in various cell types across CNS diseases. This study primarily focuses on neuronal ferroptosis, while the role of ferroptosis in other cell types following SCI requires further investigation. Lastly, our study did not include a control group of Syn Cre mice expressing wild-type *Trim32* which could present certain limitations to our findings.

## Supplementary Material

Supplementary figures.
