# Supplementary material for: TRIM32 promotes neuronal ferroptosis by enhancing K63-linked ubiquitination and subsequent p62-selective autophagic degradation of GPX4
Source: Int J Biol Sci. 2025 Jan 20;21(3):1259–74. doi: 10.7150/ijbs.106690 (PMC11781169; doi:10.7150/ijbs.106690)
Supplement: Supplementary file 1 — Supplementary figures. [file ijbsv21p1259s1.pdf]

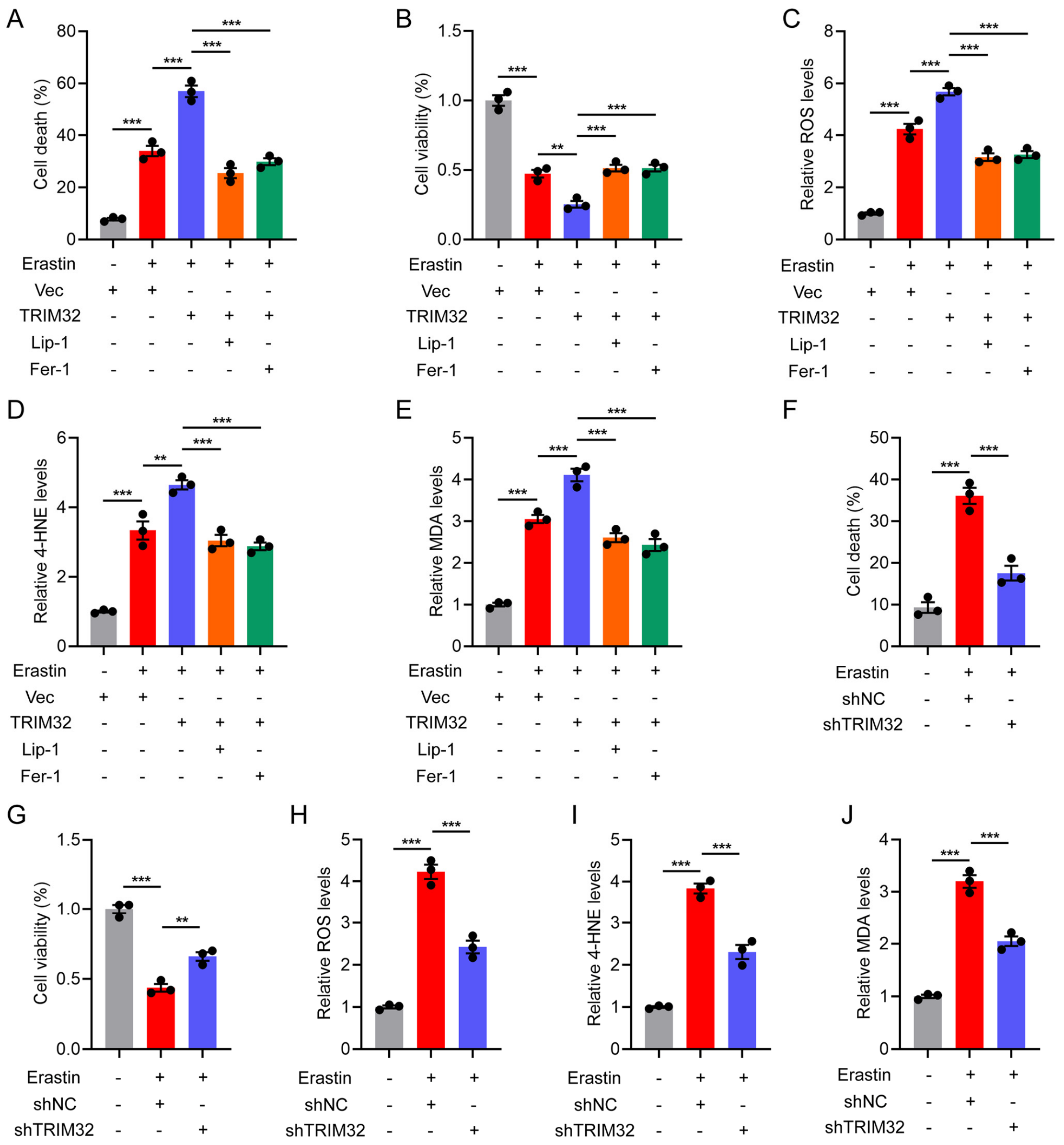

Figure S1. TRIM32 promotes Erastin-induced neuronal ferroptosis *in vitro.* (A) Cell death was assessed using Live/Dead staining and the PI positive primary neurons were quantified in vector or TRIM32-overexpression groups exposed to Erastin (5μM) for 6 hours in combination with Lip-1 or Fer-1. (B-E) Cell viability (B), ROS (C), 4-HNE (D) and MDA (E) levels were assessed in vector or TRIM32-overexpression groups exposed to Erastin (5μM) for 6 hours in combination with Lip-1 or Fer-1. (F) Cell death was assessed using Live/Dead staining and the PI positive primary neurons were quantified in shNC or shTRIM32 groups exposed to Erastin (5μM) for 6 hours. (G-J) Cell viability (G), ROS (H), 4-HNE (I) and MDA (J) levels were assessed in shNC or shTRIM32 groups exposed to Erastin (5μM) for 6 hours.

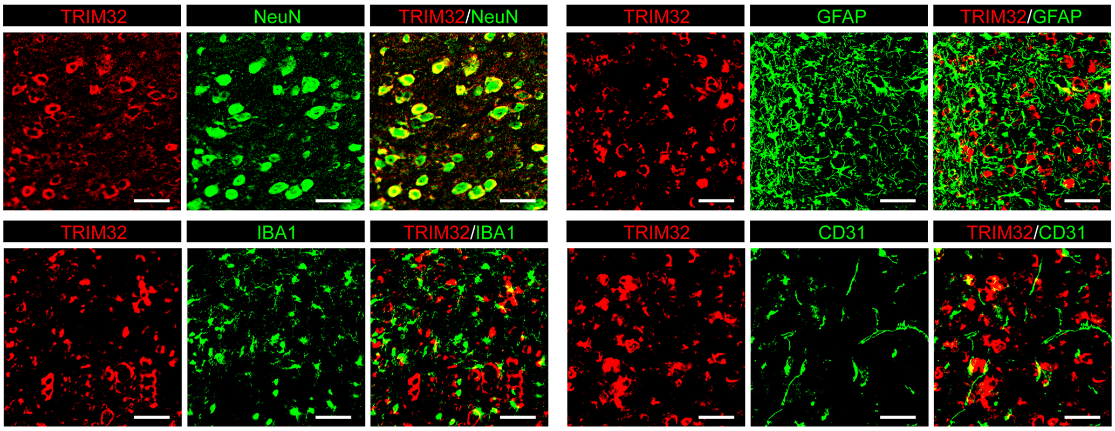

Figure S2. TRIM32 was mainly expressed in neurons in spinal cord after SCI. Representative images of co-immunofluorescence analysis of TRIM32 with NeuN (neuron maker), GFAP (astrocyte marker), IBA (microglia/macrophage marker) and CD31 (endothelial cell marker) after SCI.

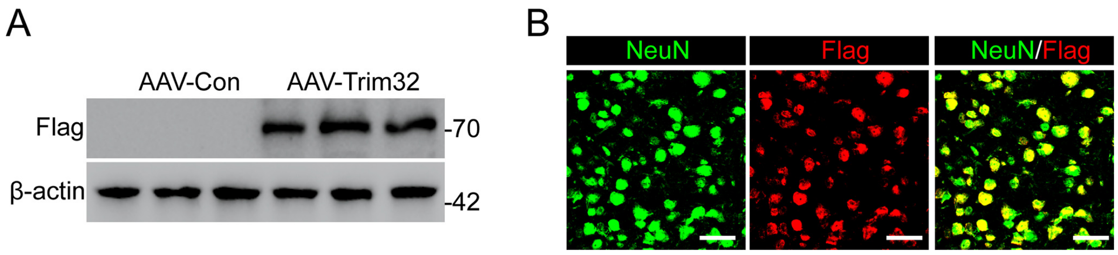

Figure S3. Transfection efficiency of AAV-PHP.eB-hSyn-Trim32 in mice. (A) Representative immunoblotting of the Flag (TRIM32) protein level in the spinal cord. (B) Representative immunofluorescence images for identifying AAV-Trim32 transfection and neuronal cell specificity. Scale bar = 100 μm.

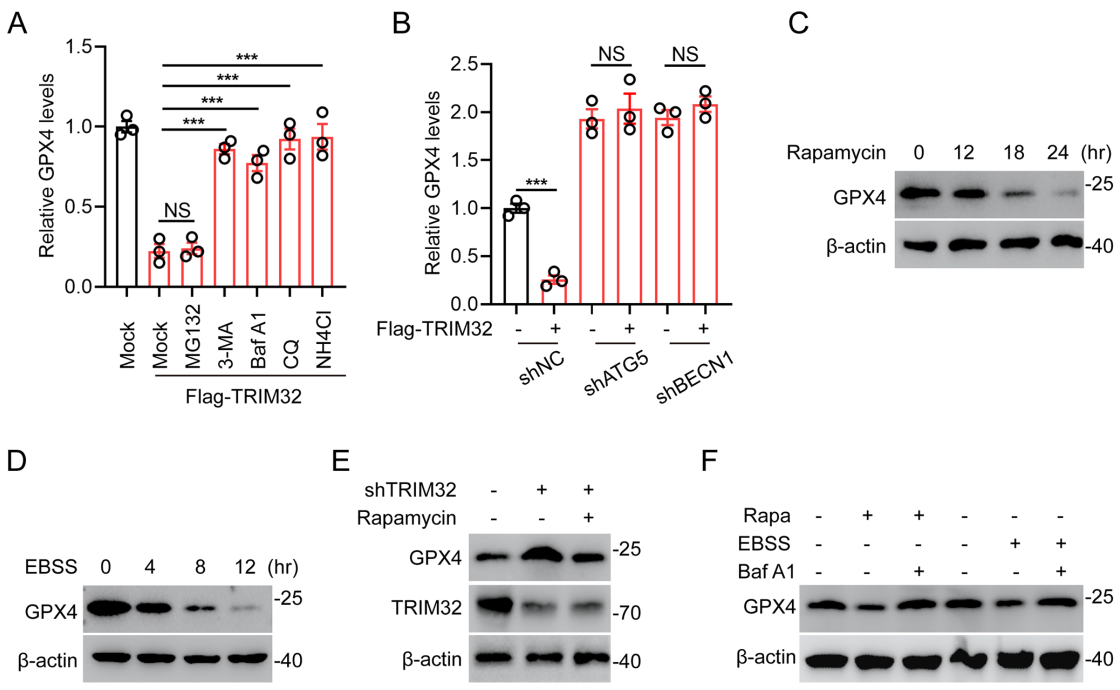

Figure S4. TRIM32 promoted autophagic degradation of GPX4. (A) Quantification of Figure 4H. (B) Quantification of Figure 4I. (C-D) Primary neurons were treated with Rapamycin (250 nM, C) or EBSS (D) with indicated time points, and the proteins were harvest for immunoblot analysis. (E) Primary neurons transfected with shNC and shTRIM32 were treated with Rapamycin (250 nM), and the proteins were harvest for immunoblot analysis. (F) Primary neurons were treated with Rapamycin (250 nM) for 24 hours or cultured with EBSS for 12 hours, in the presence or absence of Baf A1 (0.2 μM) for 6 hours. The lysates were analyzed with immunoblotting. Data were evaluated using two-tailed unpaired Student's *t*-test (B) and one-way ANOVA followed by post-hoc Bonferroni correction (A).

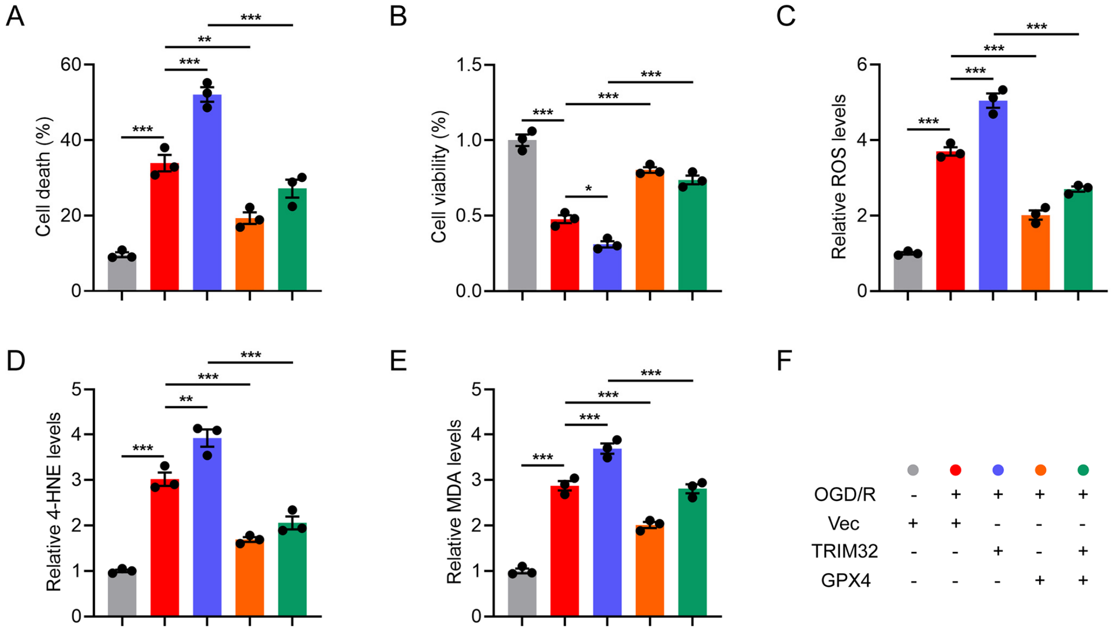

Figure S5. TRIM32 promoted neuronal ferroptosis by destabilizing GPX4 *in vitro*. (A) Cell death was assessed using Live/Dead staining and the PI positive primary neurons were quantified in indicated groups exposed to OGD/R for 2 hours. (B-E) Cell viability (B), ROS (C), 4-HNE (D) and MDA (E) levels were assessed in indicated groups exposed to OGD/R for 2 hours. Data were evaluated using one-way ANOVA followed by post-hoc Bonferroni correction (A-E).

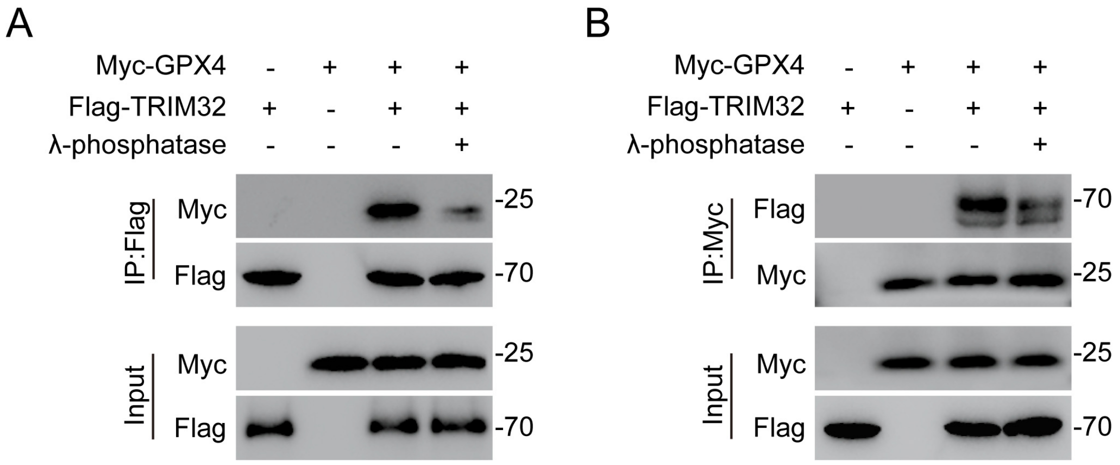

Figure S6. ROS-ATM-Chk2 signaling pathway phosphorylates TRIM32 and forms a positive feedback loop. (A-B) Co-IP assays were carried out to investigate the interaction between exogenous TRIM32 and GPX4 in the absence or presence of λ-phosphatase in HEK293T cells.

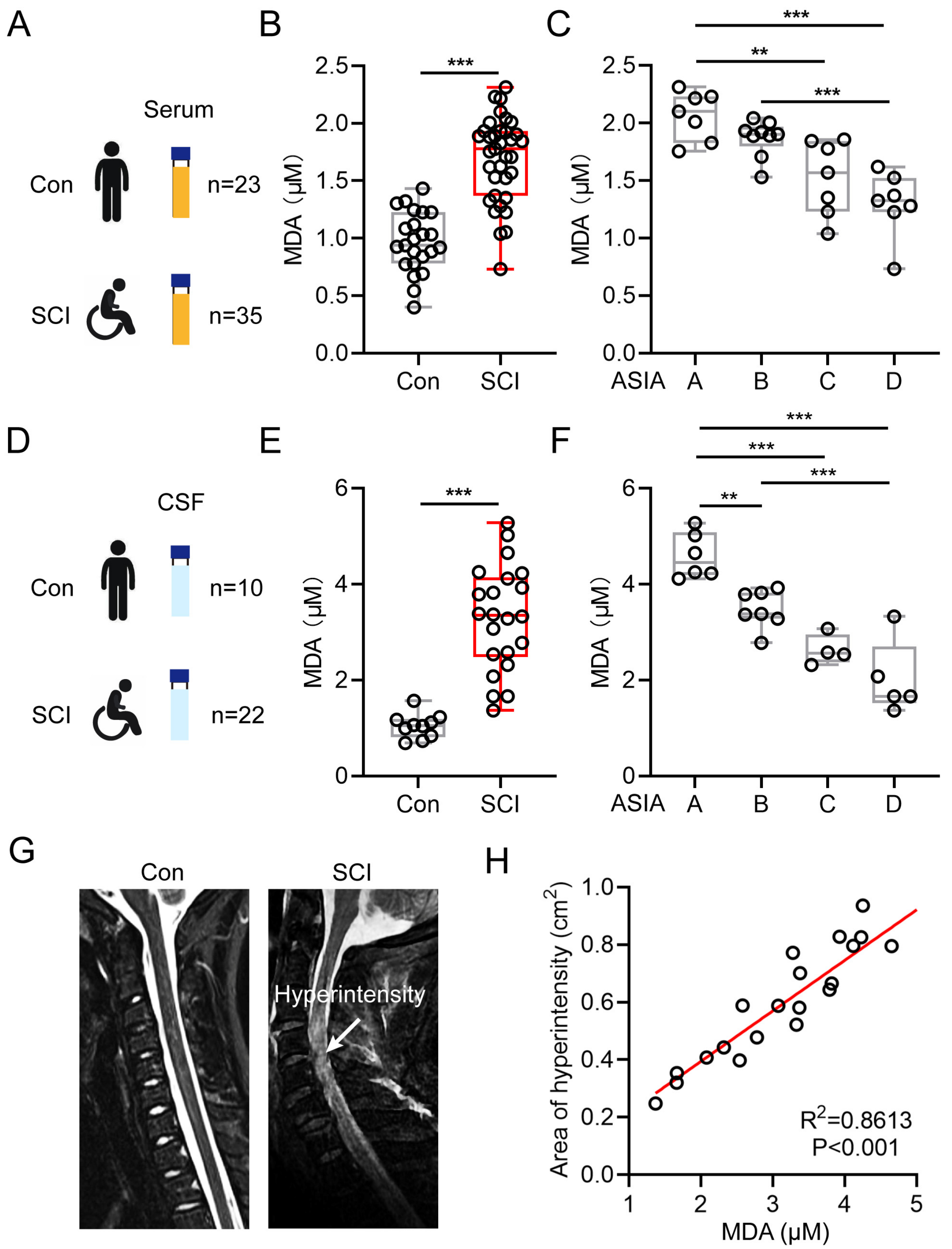

Figure S7. Clinical high serum and cerebrospinal fluid lipid peroxidation levels positively correlated with poor neurological function in patients with SCI. (A-B) Serum samples were intravenously collected and MDA levels were measured in controls (n = 23) and patients with SCI (n = 35). (C) Serum MDA levels from patients with SCI with different ASIA grades (n = 35). (D–E) CSF samples were collected via lumbar puncture and MDA levels were measured in controls (n=10) and patients with SCI (n = 22). (F) CSF

MDA levels from patients with SCI with different ASIA grades (n = 22). (G) Representative T2-weighted MRI images from healthy control and patients with SCI. Arrow indicates hyperintensity area. (H) Correlation analysis of hyperintensity area in T2-weighted MRI images and CSF MDA levels from patients with SCI (n = 22). Data were evaluated using two-tailed unpaired Student's *t*-test (B and E), one-way ANOVA followed by post-hoc Bonferroni correction (C and F), and Pearson correlation analysis (H).
